# Metabolomic Profiling in the Characterization of Degenerative Bone and Joint Diseases

**DOI:** 10.3390/metabo10060223

**Published:** 2020-05-29

**Authors:** Katherine R. Swank, Jamie E. Furness, Erin A. Baker, Corinn K. Gehrke, Stephen P. Biebelhausen, Kevin C. Baker

**Affiliations:** 1 Departments of Orthopaedic Surgery and Research, Beaumont Health, Royal Oak, MI 48073, USA; katherine.swank@beaumont.org (K.R.S.); jamie.furness@beaumont.org (J.E.F.); corinn.gehrke@beaumont.org (C.K.G.); stephen.biebelhausen@my.rfums.org (S.P.B.); kevin.baker@beaumont.org (K.C.B.); 2 Department of Orthopaedic Surgery, Oakland University William Beaumont School of Medicine, Rochester, MI 48073, USA

**Keywords:** metabolomics, arthritis, inflammatory arthropathy, small molecule metabolite synovial fluid, arthritis biomarker

## Abstract

Osteoarthritis and inflammatory arthropathies are a cause of significant morbidity globally. New research elucidating the metabolic derangements associated with a variety of bone and joint disorders implicates various local and systemic metabolites, which further elucidate the underlying molecular mechanisms associated with these destructive disease processes. In osteoarthritis, atty acid metabolism has been implicated in disease development, both locally and systemically. Several series of rheumatoid arthritis patients have demonstrated overlapping trends related to histidine and glyceric acid, while other series showed similar results of increased cholesterol and glutamic acid. Studies comparing osteoarthritis and rheumatoid arthritis reported elevated gluconic acid and glycolytic- and tricarboxylic acid-related substrates in patients with osteoarthritis, while lysosphingolipids and cardiolipins were elevated only in patients with rheumatoid arthritis. Other bone and joint disorders, including osteonecrosis, intervertebral disc degeneration, and osteoporosis, also showed significant alterations in metabolic processes. The identification of the molecular mechanisms of osteoarthritis and inflammatory arthropathies via metabolomics-based workflows may allow for the development of new therapeutic targets to improve the quality of life in these patient populations.

## 1. Introduction

The incidence of degenerative joint diseases, which impact socioeconomic and quality of life status, continues to grow worldwide; however, the root causes driving this increase are not fully elucidated. Zhao et al. reported that osteoarthritis (OA) alone affects 10% of the population, with a healthcare cost of $1778 annually per affected individual and $189 of lost wages per person per year, resulting in national excess estimated costs of $45 billion and $1.7 billion, respectively [1]. Additionally, a recent observational study by McGrath et al. demonstrated that “rheumatism or arthritis”, as a health condition category, was associated with the second highest disability-adjusted life years regardless of race or ethnicity in older Americans [2].

The initial diagnosis of degenerative joint disease is typically followed by a period of medical management of pain and dysfunction which may continue for months or years. In the case of inflammatory arthropathies, this phase of management may include biologic therapies aimed at mitigating the pro-inflammatory environment within affected joints. OA, which has traditionally been characterized as joint degeneration stemming from mechanical wear and tear, is also managed symptomatically in the early disease course. The first-line pharmacological treatment of OA consists of non-steroidal anti-inflammatories which act as cyclooxygenase inhibitors. When conservative management is unsuccessful, surgical treatment may be pursued in the setting of end-stage osteo- or inflammatory arthritis. Affected joints may be replaced (e.g., total joint arthroplasty, interpositional arthroplasty), excised (resection arthroplasty), or fused (arthrodesis). Despite surgical options which may restore some degree of function and provide pain relief, these treatments are inferior to a native joint due to limitations (e.g., reduced range of motion, wear debris generation over the lifecycle of total joint arthroplasty) and complications (e.g., postoperative infection, fracture). The treatment of inflammatory arthropathies, including rheumatoid arthritis (RA) and systemic lupus erythematosus (SLE), has improved due to the development and use of biologic therapies targeting the inflammatory signaling cascade. While these treatments have resulted in improved quality of life and slowed disease progression and have delayed or eliminated surgical interventions in patients with inflammatory arthropathies, similar disease-modifying agents are not currently available for the treatment of OA.

Metabolomics-based research has improved our understanding of the mechanisms associated with a variety of pathologic states. Applying metabolomics to the diagnosis and characterization of degenerative joint diseases may provide insight into the molecular basis underlying the various disease processes leading to joint degeneration. The development of these new therapeutic strategies is predicated on understanding the relevant tissue, cellular, and molecular processes related to the disorder.

## 2. Osteoarthritis

Osteoarthritis, including “primary” and “secondary” OA, is the most common type of arthritis in the United States, affecting approximately 25% of the patient population with arthritic conditions [3]. Primary OA is idiopathic and commonly described in purely mechanical terms as “wear and tear” arthritis. Secondary OA includes joint degeneration with a known cause, such as ACL rupture-induced post-traumatic osteoarthritis (PTOA), which leads to joint degeneration. Although PTOA-associated joint degeneration has historically been ascribed to the alteration of normal joint biomechanics, more recent evidence has demonstrated the role of biology in the onset and progression of PTOA. Treatments for primary and secondary OA are still symptomatically driven, including the use of systemic anti-inflammatory agents, activity modification, and local injections of corticosteroids or hyaluronic acid-replacing agents. Disease-modifying osteoarthritic drugs (DMOADs), which are currently in development with promising preliminary data, may provide the greatest impact in early-stage arthritic disease before significant joint degradation occurs. The use of metabolomics-based techniques to detect the onset and progression of OA may lead to novel targeted therapies, including DMOADs (Table 1).

### 2.1. Local Biomarkers of Osteoarthritis

#### 2.1.1. Bone

Alterations in subchondral bone, which may occur in late-stage OA, have been described as a result of the altered mechanical loading of the underlying bone due to the progressive degradation of articular cartilage. Yang et al. compared metabolite profiles in centric subarticular spongiosa harvested from the medial femoral condyle in 42 patients with primary knee OA who were undergoing joint arthroplasty [5]. Metabolite profiles were compared with centric subarticular spongiosa harvested from the lateral femoral condyle using an ultraperformance liquid chromatography-quadrupole time-of-flight mass spectrometry (UPLC/Q-TOF-MS) workflow [5]. In this series, the role of the taurine and hypotaurine metabolism was observed; specifically, taurine levels were greater in sclerotic subchondral bone samples [5]. Taurine supplementation has been shown to both promote the osteoblastic differentiation of human mesenchymal stem cells via the extracellular signal-regulated kinase (ERK) pathway and inhibit osteoclastic differentiation [12,13]. Briggs et al. used matrix-assisted laser desorption/ionization (MALDI-MS) imaging to characterize the spatial distribution of N-linked glycans, which are associated with numerous biologic functions (e.g., cell chemotaxis, protein-folding) within sclerotic tibial subchondral bone from three patients with OA [14]. A combination of MALDI-MS imaging and liquid chromatography-electrospray ionization-mass spectrometry (LC-ESI-MS) identified similar N-glycan species between the subchondral bone and the overlying articular cartilage [14]. Recently, high-mannose N-glycan species have also been associated with OA progression in humans and mice [15].

#### 2.1.2. Synovial Fluid

Synovial fluid represents a matrix in direct proximity to the degenerative process which may be sampled and analyzed to provide mechanistic and diagnostic information. This protein-rich ultrafiltrate from plasma lubricates the articular cartilage-covered surfaces of a joint via non-Newtonian properties and contains both hyaluronic acid and lubricin (protein; proteoglycan 4) for boundary lubrication. Mickiewicz et al. analyzed synovial fluid from OA joints and reported 11 key metabolites with altered concentrations in arthritic joints; specifically, perturbations in the tricarboxylic acid (TCA) cycle and a shift towards anaerobic metabolic pathways were observed [6]. An analysis of synovial fluid samples from knee OA patients by Kim et al. showed that more severe radiographic evidence of arthritis (Kellgren–Lawrence grades 3 and 4) was associated with a shift toward fatty acid metabolism, which may reflect the initiation of downstream pathologic processes centered around energy conservation, such as autophagy [4]. Similarly, Jonasdottir described increased fatty acids in the synovial fluid of patients with OA [16]. Using electrospray ionization (ESI) and liquid chromatography-mass spectrometry (LC-MS) to analyze synovial fluid samples for sphingolipid species in post-mortem (control), RA, and OA patients, Kosinska et al. identified 19 sphingomyelin species associated with branched fatty acids, which may be linked to stress response regulation, apoptosis, and senescence [4,17]. SM34:1 was the predominant sphingomyelin species identified in this series, and the overall species were 2.4-fold greater in early OA and 4.4-fold greater in late OA [17]. Ceramide, which has pro-inflammatory and apoptotic effects, also demonstrated increased levels based on the severity of OA, with levels 2-fold greater in early OA and 3.9-fold greater in late OA [17]. In a study of disease chronicity, Carlson et al. identified shifts in synovial fluid metabolites based on the duration of OA diagnosis [18]. This series showed two distinct phenotypes of OA: one group exhibited degeneration that was inflammatory-based and progressed to oxidative stress, while a second group demonstrated features associated with the structural degradation of tissue [18]. In the first group, inflammatory markers included butyrate and leukotrienes, while markers associated with glutathione metabolism emerged at later timepoints, indicating a transition from inflammation to oxidative stress as a driving mechanism for joint degeneration [18]. The second group was associated predominately with structural degradation-associated features, including alterations in the glycosaminoglycan (GAG) metabolism, tryptophan metabolism, and ascorbate degradation [18]. An altered GAG metabolism may reflect the direct catabolism of normally GAG-rich articular cartilage, while the tryptophan metabolism in the context of joint diseases has been associated with the inflammation and expansion of pro-inflammatory T-lymphocyte populations, including T_h_17 cells [19]. In a series of 25 patients with OA, Zheng et al. analyzed synovial fluid extracted in the setting of total knee arthroplasty (TKA) using gas chromatography time-of-flight mass spectrometry (GC-TOF/MS), and showed six metabolites strongly associated with OA, including glutamine, 1,5-anhydroglucitol, gluconic lactone, tyramine, threonine, and 8-aminocaprylic acid [20]. Increased gluconic lactone may be related to autoxidation from high levels of reactive oxygen species in the joint, which may degrade cartilage through the activation of metalloproteinases as well as accumulate lipid peroxidation products. Differential gluconic lactone concentrations between OA and RA patients may also differentiate the distinctive etiologies of arthritis.

### 2.2. Systemic Biomarkers of Osteoarthritis

The assessment of serum specimens has also implicated fatty acid metabolism in the development of OA. Senol et al. reported increased levels of multiple phospholipids involved in the upregulation of neutrophil activation, as well as increased propane-1,3-diol, which is associated with the glycerolipid metabolism [7]. In a series of 72 patients, Zhang et al. found that arginine, which inhibits cathepsin K and B isoforms, was significantly depleted in the plasma of patients with arthritic knees [8]. Further investigation demonstrated that ornithine and proline concentrations were also 2.2 and 1.2 times greater, respectively, in OA patients compared to controls, implicating an imbalance between cartilage repair and degradation associated with the ornithine pathway [8]. While arginine was the most significant metabolite in this study, phosphatidylcholine derivatives and hydroxysphingomyeline, which are involved in signal transduction and membrane trafficking, were also significantly lower in patients who had undergone TKA and, therefore, may play a significant role in OA [8]. Zhai et al. described the branched chain amino acid (BCAA) to histidine ratio as a biomarker of knee arthritis, and hypothesized that increased BCAAs may be associated with the release of amino acids from collagen breakdown [9]; however, in an ovine model of arthritic changes induced via meniscal destabilization or anterior cruciate ligament (ACL) transection, Maher et al. reported increased serum dimethyl sulfone after meniscal destabilization and 3-methylhistidine, but decreased BCAAs after ACL transection [21]. Both studies observed increased levels of lactate, indicating a transition to anaerobic metabolism and oxidative stress [9,21]. In a study of urinary metabolites associated with the radiographic progression of knee OA, Loeser et al. found that advanced OA was associated with greater levels of interleukin-6 (IL-6), which is indicative of systemic inflammation [10]. In the group of OA progressors at 18 month follow-up, urinary concentrations of the carboxylic acid hippurate were increased and accompanied by a decreased concentration of trigonelline, which the authors suggest may implicate the role of the gut microbiome in osteoarthritis progression.

## 3. Inflammatory Arthropathies

Rheumatoid arthritis and other inflammatory arthropathies are autoimmune diseases attributed to a combination of genetic and environmental factors. RA is a T-cell major histocompatibility complex (MHC) II-mediated immune response against soft tissues, cartilage, and, eventually, bone. Ankylosing spondylitis (AS) is a seronegative spondyloarthropathy associated with human leukocyte antigen B27 (HLA-B27), a MHC I allele; specifically, 90–95% of patients with AS are HLA-B27 positive [22]. In the early stages of disease, anti-inflammatory (e.g., non-steroidal anti-inflammatory medications) and steroid-based therapies may be used to reduce pain and inflammation, while disease-modifying agents are used to delay disease progression. Major proinflammatory cytokines (e.g. interferon-gamma (IFN-γ); interleukin-1beta (IL-1β); IL-6; and tumor necrosis factor-alpha (TNF-α) IFN-γ, IL-1β, IL-6, and TNF-α), are implicated in the inflammatory cascade leading to joint damage and are targets for disease-modifying agents and biologics. Metabolomics-based characterization may provide new information regarding the molecular basis of the RA disease process which may lead to improvement of current biologics and the development of additional targeted therapeutic strategies (Table 2). As in OA, surgical treatment for specific joint involvement represents the final phase of intervention in advanced disease.

### 3.1. Local Biomarkers of Inflammatory Arthropathies

#### Synovial Fluid

In a small series of RA patients and healthy controls, Carlson et al. conducted a global metabolomic analysis of synovial fluid via normal phase LC-MS which showed alterations in amino acid metabolism, leukotriene biosynthesis, alpha linolenic acid metabolism, and glucocorticoid and mineralocorticoid metabolism, as well as steroid biosynthesis [23]. In a study of global and targeted metabolomic analyses of OA and RA patients with healthy controls, 1,5-anydroglucitol, threonine, glutamine, and gluconic acid were significantly increased in the synovial fluid of RA patients [20]. Gluconic acid was elevated in RA patients compared with OA patients [20]. In a series of five RA patients undergoing knee arthroscopy, Giera et al. used capillary liquid chromatography with tandem mass spectrometry (LC-MS/MS) characterization to assess lipid and lipid mediator profiles and found increased leukotriene B4 (LTB4) isomer in high concentrations; deuterated leukotriene B4 (LTB4d4); deuterated prostaglandin-E2 (PGE2d4); docosahexaenoic acid (DHA, or FA22:6); and eicosapentaenoic acid (EPA, or FA20:5), which is lipoxygenase 5 (LOX5-) and lipoxygenase 15 (LOX15-)-generated [32]. The pattern of the expression of these lipid mediators indicates the persistent activation of resolution pathways, which is unexpected given the chronic inflammatory state within the joints of RA patients. Kosinska et al. performed a phospholipid-based analysis of early OA, late OA, RA, and control patients using electrospray ionization tandem mass spectrometry (ESI-MS/MS), and found elevated sphingomyelins and ceramides in synovial fluid samples from both OA and RA patients; however, lysosphingolipids differed between OA and RA, with the elevation of SPH d18:0 and SPC d18:0 in RA only [33]. Similarly, cardiolipins were elevated only in the synovial fluid of RA patients [33]. In a follow-on study focusing on sphingolipids, which may affect the inflammation of the synovium and subsequent native repair response, a range of 21 to 50 lipid species differed between the cohorts [17]. Using a proton nuclear magnetic resonance (^1^H-NMR) spectroscopy approach, Anderson et al. identified 32 metabolites which differed significantly between cohorts of OA and RA patients; predominately, these metabolites were related to amino acid synthesis, taurine metabolism, glycophospholipid metabolism, glycolysis, and the TCA cycle [34]. Specifically, synovial fluid from OA patients showed significantly greater glycolytic- and TCA-related substrates, indicating greater anaerobic cellular metabolism in RA compared to OA [34]. The lack of substrates due to glycolysis-reliant immune cells leads to a hypoxic environment, resulting in the synovial proliferation characteristic of RA [34]. Using a combined proteomic and metabolomic approach, Yang et al. analyzed synovial fluid from 25 RA patients and 10 controls undergoing synovectomy or total joint replacement, and reported significantly increased β-mannosylglycerate, carnitine, diglycerol, lactic acid, and pipecolinic acid in the RA cohort, while 5-methoxytryptamine, citric acid, gluconic lactone, D-glucose, glucose-1-phosphate, mannose, ribitol, and L-valine were significantly decreased in these patients [24].

### 3.2. Systemic Biomarkers of Inflammatory Arthropathies

Using gas chromatography-mass spectrometry (GC-MS) to analyze serum samples from RA patients, Zhou et al. reported increased levels of fatty acids and cholesterol as well as decreased levels of amino acids and glucose compared to controls [27]. Andonian et al. analyzed the serum samples of patients with RA for cytokines using ELISA and for organic acids and lipids using GC-MS and NMR [35]. They reported a positive correlation between plasma miR143 and systemic inflammation, specifically plasma IL-6 and interleukin-8 (IL-8) [35]. In a similar approach using GC-MS in combination with LC-MS, blood samples from patients with RA showed increased levels of glyceric acid, D-ribofuranose, and hypoxanthine; by contrast, histidine, threonic acid, methionine, cholesterol, asparagine, and threonine decreased [29]. In a series of pre-symptomatic RA patients, plasma samples were analyzed using a LC-MS and lipid profiling approach and showed elevated levels of lysophospatidylcholines and tryptophan metabolites, as well as reductions in acyl-carnitines and fatty acids [26]. In another study of plasma from patients with RA analyzed via LC-MS, increased levels of arginine, aspartic acid, glutamic acid, glycine, isoleucine, leucine, phenylalanine, serine, threonine, and valine were found, along with decreased levels of betaine, lysine, and methyl nicotinamide [28]. Like Andonian et al., Lauridsen et al. used ^1^H-NMR to assess plasma serum samples from patients with RA and demonstrated that cholesterol, lactate, acetylated glycoprotein, and phosphatidyl choline were elevated, while high-density lipoproteins (HDLs) were decreased [30]. These altered cholesterol levels were indicative of increased inflammation, while increased lactate production was linked to anaerobic metabolism, which may indicate increased oxidative stress [30].

The evaluation of blood and urine samples from patients with RA using capillary electrophoresis time-of-flight mass spectrometry (CE-Q-TOFMS) indicated decreased levels of histidine, methionine, guanidoacetic acid, and serine, as well as increased levels of glyceric acid, phenylalanie, hypotaurine, and tyrosine [31]. The identified metabolites are involved in several metabolic pathways, including glycolysis, the TCA cycle, and amino acid metabolism; specifically, plasma histidine and guanidoacetic acid as well as urine hypotaurine were closely associated with RA disease activity [31]. Several of these metabolite (i.e., histidine, glyceric acid) trends overlapped with the Madsen et al. series. Li et al. analyzed serum samples from patients with RA and Sjögren’s syndrome as well as respective healthy controls, using ultra-high-performance liquid chromatography coupled with high-resolution mass spectrometry (UPLC-HRMS) [25]. Compared to both Sjögren’s syndrome and control samples, the RA samples showed increased levels of 4-methoxyphenylacetic acid, glutamic acid, L-leucine, L-phenylalanine, L-tryptophan, L-proline, glyceraldehyde, fumaric acid, and cholesterol; however, capric acid, argininosuccinic acid, and bilirubin were all decreased in patients with RA [25]. Further, 4-methoxyphenylacetic acid, L-leucine, and L-phenylalanine were isolated as a three-metabolite panel of RA-specific biomarkers [25]. Increased cholesterol and glutamic acid were reported by several studies, including Zhou et al., Lauridsen et al., and Smolenska et al. [27,28,30].

## 4. Other Musculoskeletal Conditions

### 4.1. Osteonecrosis/Avascular Necrosis

Osteonecrosis, an idiopathic process affecting predominately males and involving bilateral hips in 80% of cases, has known multifactorial associations, including irradiation, trauma, hematologic conditions, marrow-replacing diseases, sickle cell disease, alcoholism, and hypercoagulable states; therefore, the implicated molecular mechanisms may be as varied as the causes. Yang et al. performed the urinary metabolomic profiling of a patient cohort with confirmed femoral osteonecrosis (with varied histories, including alcohol consumption, femoral neck or intertrochanteric fracture, corticosteroid therapy, and idiopathic osteonecrosis) and found increased urea, deoxycholic acid, and phosphatidylethanolamine [36]. In a plasma-based metabolomic analysis of patients with confirmed osteonecrosis, Liu et al. reported alterations in lipid metabolism, nucleotide metabolism, and cysteine metabolism; lipid metabolism was primarily downregulated [37]. Using the same analytical approach applied to a series of patients with avascular necrosis (AVN), Narayanan et al. identified increased levels of methionine and homocysteine, while betaine, B12, and B6 were decreased [38]. Zhu et al. analyzed the bone-trabeculae of the osteonecrotic femoral head and showed that amino acids were affected more profoundly than lipids, nucleotides, or pyrrolidines in these local tissues when compared to non-osteonecrotic control patients [39]. In a rabbit model of corticosteroid-induced AVN, a plasma-based metabolomic algorithm was again applied and showed that phosphatidyl ethanolamine and phosphatidyl choline were decreased, while lipid metabolism was inhibited in early-stage necrosis (i.e., 1- to 3-week timepoints vs. “late” 6-week verification timepoint), prior to behavioral changes or imaging-based detection [40]. Ren et al. further connected these results to prior work by Tian et al., describing the cascading effects of phospholipid anabolism, leading to bone cell cytomembrane instability followed by apoptosis and, ultimately, osteonecrosis [40,41].

### 4.2. Intervertebral Disc Degeneration

The degeneration of the intervertebral disc remains one of the leading causes of low back pain. While definitive surgical management commonly involves the removal of the disc and fusion of adjacent vertebrae, new biologic therapies aimed at halting or reversing disc degeneration are emerging. Shan et al. surveyed metabolite levels in the plasma of patients with lumbar disc herniations using GC-MS and found that glutamic acid, aspartic acid, and glycine levels were elevated while glucose 1-phosphate was decreased [42]. Radek et al. utilized proton high resolution-magic angle spinning nuclear magnetic resonance (^1^H-HR-MAS-NMR) spectroscopy to characterize metabolite levels within intervertebral disc tissue removed from patients at the time of surgery for MRI-confirmed disc degeneration [43]. A subsequent analysis showed that, compared to mild degeneration (Pfirrmann grade III discs), more severe degenerative changes (Pfirrmann grades IV and V) were associated with increased concentrations of creatine, glycine, hydroxyproline, alanine, leucine, valine, acetate, isoleucine, α,β-glucose, and myoinositol, but decreased levels of chondroitin sulfate [43]. Decreased chondroitin sulfate is likely a reflection of decreased sulfated glycosaminoglycans within the degenerated nucleus pulposus of the disc, while increases in hydroxyproline may reflect an alteration in the overall collagen content and organization within the annulus fibrosus.

### 4.3. Osteoporosis 

Osteoporosis is a common bone metabolic disorder resulting in a loss of bone density, culminating in a significantly increased risk of fracture. Primary osteoporosis is age-related and occurs in the absence of underlying disease, while the causes of secondary osteoporosis include endocrine disorders (e.g., glucocorticoid-induced, hyperthyroidism, hypogonadism, diabetes mellitus, growth hormone deficiency), gastrointestinal disorders (e.g., celiac disease, inflammatory bowel disease, anorexia nervosa), hepatic disorders (e.g., hemochromatosis, chronic liver disease), hematologic disorders (e.g., multiple myeloma), renal disorders (e.g., renal tubular acidosis, chronic kidney disease), autoimmune disorders (e.g., RA, SLE, AS, multiple sclerosis), and iatrogenic causes (e.g., pharmacologic-induced, gastric bypass surgery) [44]. In a rat model of estrogen deficiency-related osteoporosis, Ma et al. used GC-TOF/MS to identify systemic metabolite patterns in plasma samples and observed increased fatty acids, including arachidonic acid, which is associated with increased osteoclastogenesis [45]. Additionally, metabolites associated with the structure of collagen (hydroxyproline) and ketone bodies (3-hydroxybutyric acid) were also increased in rats with estrogen-associated bone loss [45]. 3-hydroxybutyric acid (3HB) has been shown to improve preosteoblast differentiation and mineralization. While osteoporosis is thought to be a condition where osteoclast-mediated bone resorption outpaces osteoblast-mediated bone formation, it is now widely recognized that there is a considerable deficit in differentiation and mineralization activities in osteoblasts from osteoporotic individuals. A lack of serum 3HB may reflect this degenerative state of osteoblasts in osteoporosis.

In a series of pre- and post-menopausal Chinese women, Miyamoto et al. used capillary electrophoresis-mass spectrometry (CE-MS) to quantify circulating metabolites associated with bone density [46]. Hydroxyproline was elevated in the group of patients with a lower bone mineral density, which corresponded with preclinical work by Ma et al., while glycylglycine and cystine were significantly decreased [45,46]. Pontes et al. utilized ^1^H-NMR spectroscopy to differentiate between osteopenic and osteoporotic patients [47]. Patients with osteoporosis had higher concentrations of cholesterol, leucine and isoleucine, and lactate and unsaturated lipids, while osteopenic bone showed increased levels of allantoin, a biomarker of oxidative stress [47].

## 5. Conclusions

The characterization of local and systemic metabolites provides value in mechanistic studies of pathology. Analytical techniques used in each study were dictated by the tissue matrix of interest, which may introduce variability in the identification of pathologic metabolite patterns. The continued investigation of the patterns of metabolite expression in fluid and tissue matrices generates important information regarding bone and joint disorders. Metabolomic-based research may drive the development of new diagnostic strategies to facilitate the early detection of tissue degeneration and joint dysfunction as well as provide insight into therapeutic targets that may slow or halt disease processes, ultimately improving the quality of life in patient populations affected by musculoskeletal conditions.

## Figures and Tables

**Table 1:** Metabolomics-based studies of osteoarthritis.

Author	Year	Specimen Analysis	Upregulated Metabolites	Downregulated Metabolites	Pathways Affected
Kim, S [4]	2017	Location: Knee Matrix: Synovial Fluid Characterization: CG/TOF MS	Metabolites discriminating early vs. late stage OA: Squalene; Palmitoleic acid; Pentadecanoic acid; Glycerol; Myristic acid; Lignoceric acid; Alpha-tocopherol; Heptadecanoic acid; Oleic acid; Linoleic acid; Threose; 3-Hydroxypropionate; Lanosterol; Ethanolamine; Putrescine; N-Carbamoylaspartate; Capric acid; Malate; Asparagine; Arachidonic acid; Pelargonic acid; Benzoate; Palmitic acid; 1-Monostearin; Salicylaldehyde; Stearic acid; Adipate; Phenylalanine	N/A	Glycolysis; TCA cycle; Amino acid metabolism; Fatty acid metabolism; Glycerolipid metabolism; Glycerophospholipid metabolism
Yang, G [5]	2016	Location: Knee Matrix: Subchondral bone Characterization: UPLC/Q-TOF-MS	Taurine; L-Tyrosine; Hypoxanthine; L-Carnitine; Uridine; Guanosine; 2-Hydroxycinnamic acid; Triethanolamine; 2-Phenylacetamide; Octadecylamine; Retinol acetate	Trichocarposide; Lyso PC(P-16:0); Lyso PC(18:1(9Z)); N6,N6,N6-trimethyl-L-Lysine; 5-(4′-Hydroxyphenyl)-Gamma-Valerolactone-4′-O-Glucoronide; SM(d18:1/22:1(13Z)); SM(d16:1/24:1(15Z))	Taurine and hypotaurine metabolism; β-Alanine metabolism; Phenylalanine metabolism; Tyrosine metabolism; Lysine degradation; Pyrimidine metabolism; Purine metabolism; Sphingolipid metabolism
Mickiewicz, B [6]	2015	Location: Knee Matrix: Synovial Fluid Characterization: ^1^H-NMR; GC-MS	Fructose; Citrate	O-Acetylcarnitine; N-Phenylacetylglycine; Methionine; Ethanol; Creatine; Malate; Ethanolamine; 3-Hydroxybutyrate; Hexanoylcarnitine	TCA cycle; Fatty acid and lipid metabolism
Senol, O [7]	2019	Location: Systemic Matrix: Serum Characterization: LC/Q-TOF/MS/MS	PA (18:2(9Z,12Z)); PA (16:0/16:0); Phosphatidylethanol-amine; Propane-1,3-diol sulfate; Benzoic acid; Phosphoric acid; Butyric acid; Acetic acid; L-Valine; L-Alanine; Stearic acid; Benzeneethanamine; Carbamic acid; Hydroxylamine; Indoleacetic acid; Urea; Oleic Acid; Lyso- PC(18:2(9Z,12Z))	Glycine	Ether lipid metabolism; Glycerophospholipid metabolism; Glycerolipid metabolism; Cysteine and methionine metabolism; Phenylalanine metabolism; Oxidative phosphorylation; Butanoate metabolism; Glycolysis; Gluconeogenesis; Aminoacyl-t*RNA* biosynthesis; Alanine, aspartate, and glutamate metabolism Fatty acid biosynthesis; Nitrogen metabolism; Tryptophan metabolism; Arginine and Proline metabolism; Glycerine, serine, threonine metabolism
Zhang, W [8]	2016	Location: Systemic Matrix: Serum Characterization: TQ UPLP/MS	Ornithine; Proline	Arginine; LysoPhosphatidylch-oline acyl C28:1; Phosphatidylcholine diacyl C36:6; Phosphatidylcholine acyl-alkyl C36:2; Phosphatidylcholine acyl-alkyl C38.0; Hydroxy-Sphingomyeline C14:1	Arginine catabolism
Zhai, G [9]	2010	Location: Systemic Matrix: Serum Characterization: Q TRAP; LC/MS/MS	Valine; Leucine	N/A	Arginine catabolism
Loeser, RF [10]	2016	Location: Systemic Matrix: Urine Characterization: ^1^H-NMR	Glycolate; Hippurate; Histidine	Trigonelline; Alanine; N,N-Dimethylglycine	Amino acid metabolism; Lipid metabolism; Glycosphingolipid metabolism; GalNAc β(l-3)Gal pathway
Lamers, R [11]	2005	Location: Systemic Matrix: Urine Characterization: ^1^H-NMR	Hydroxybutyrate; Pyruvate; Creatine/creatinine Glycerol	Histidine; Methylhistidine	Histidine metabolism; Fat metabolism

**Table 2:** Metabolomics-based studies of rheumatoid arthritis.

Author	Year	Specimen Analysis	Upregulated Metabolites	Downregulate Metabolites	Pathways Affected
Carlson, AK [23]	2019	Location: Not stated Matrix: Synovial Fluid Characteriz-ation: HPLC-MS	5-hydroxyibuprofen 5β-pregnane-3α, 20α-diol Traumatic acid	5-methylcytosine; Glutamyl-cysteine; Arginine; Phenylacetaldehyde; Glutamyl-cysteine; resveratrol; 5-methylcytosine; 2′-deoxyuridine; Deoxyadenosine; 2-aminoethylphosphonic acid	Ibuprofen metabolism; Gluco- and Mineralcorticoid metabolism; α-linolenic acid metabolism; Gene expression; γ-Glutamyl cycle; Biological oxidation; Arginine biosynthesis; DNA methylation; NAD metabolism; NO_2_-dependent IL-12 pathway; Pyrimidine metabolism; Arginine and Proline metabolism; Nitric oxide metabolism; VEGFR1 specific signals; Putrescine biosynthesis; Creatine biosynthesis; Arginine and ornithine metabolism; Lipoate biosynthesis; SHP2 signaling; Corticosteroids and cardioprotection; Glutathione biosynthesis; Angiotensinogen metabolism; Protein repair; Phenylethylamine degradation; Amino acid metabolism; Vitamin C metabolism; Endothelin pathways; Ion channels; Urea cycle; Wybutosine biosynthesis; Molybdenum cofactor biosynthesis; ABC transporters; A9 β1 integrin signaling; Citrulline- nitric oxide cycle; Phenylalanine degradation
Yang, XY [24]	2015	Location: Knee Matrix: Synovial fluid and synovial tissues Characteriz-ation: GC/TOF MS	β-Mannosylglycerate; Diglycerol; Lactic acid; Carnitine; Pipecolinic acid	5-Methoxytryptamine; Citric acid; Gluconic lactone; D-Glucose; Glucose-1-phosphate; Mannose; Ribitol; L-Valine	Tryptophan metabolism; Lysine degradation; Citrate cycle; Pentose phosphate pathway; Glycolysis; Fructose and mannose metabolism; Lysine degradation; Pentose and glucuronate interconversions; Valine, leucine, and isoleucine degradation
Li, J [25]	2018	Location: Systemic Matrix: Serum Characteriz-ation: UPLC-HRMS	4-Methoxyphenylacetic acid; Glutamic acid; L-Leucine; L-Phenylalanine; L-Tryptophan; L-Proline; Glyceraldehyde; Fumaric acid; Cholesterol	Capric acid; Argininosuccinic acid; Billirubin	Inflammation injury; Amino acid metabolism; Oxidative stress; Phospholipid metabolism; Cortisone metabolism; Bilirubin metabolism
Surowiec, I [26]	2016	Location: Systemic Matrix: Plasma Characteriz-ation: LC-MS	LPC(14:0),(16:0), (16:1),(18:1) (18:3),(20:4),(20:3); Phosphocholines (30:1),(32:1), (32:2),(34:2),(34:4),(O-34:3); Sphingomyelins(33:1),(32:1), (38:1),(39:1); Kynurenine; 3-Indolelactic acid; Hypoxanthine	β-Hydroxypalmitic acid; Oleic acid; Tryptophan	Increased lipid inflammation; Increased oxidative stress; Increased beta oxidation/energy demands; Tryptophan metabolism; Xanthine oxidase metabolism
Zhou, J [27]	2016	Location: Systemic Matrix: Serum Characteriz-ation: GC-MS	Eicosanoate; Docosahexaenoate; Palmitelaidate; Monostearin; *Cis*-f,8,11-Eicosatrienoate; Hexadecanoate; Arachidonate; Oleate; *Trans*--9-Octadecenoate; D-Mannose; Scyllo-Inositol; Glycerol; Ribose; Cholesterol	Glucose; Urate; Methionine; Threonine; Serine; Alanine; Leucine; Lysine; Valine; Isoleucine; Asparagine; Phenylalanine; Tyrosine; Proline; Urea; 3-Hydroxybutanoate; 2-Ketoisocaproate; 3-Methyl-2-Oxovalerate; 2-Aminobutyrate; Alanine; Pyroglutamate Trans-4-Hydroxy-D-proline; Urate; Ribonate; 1,5-Anhydrosorbitol	Fatty acid metabolism; Alanine, aspartate, and glutamate metabolism; Valine, leucine, and isoleucine metabolism; Amino acid metabolism; Glutathione metabolism; Glycine, serine, and threonine metabolism; Cysteine and methionine metabolism; Arginine and proline metabolism; Nucleotide metabolism; Glycolysis; Carbohydrate metabolism; Inositol phosphate metabolism
Smolenska, Z [28]	2016	Location: Systemic Matrix: Plasma Characteriz-ation: LC-MS/MS	Arginine; Aspartic acid; Glutamic acid; Phenylalanine; Serine; Threonine	Lysine	Amino acid metabolism; Nicotinamide metabolism
Madsen, RK [29]	2011	Location: Systemic Matrix: Serum Characteriz-ation: LC-MS	Glyceric Acid; D-Ribofuranose; Hypoxanthine	Histidine; Threonic acid; Methionine; Cholesterol; Asparagine; Threonine	Increased nucleotide synthesis; Ascorbic acid metabolism
Lauridsen, M [30]	2010	Location: Systemic Matrix: Plasma Characteriza-tion: ^1^H-NMR	Cholesterol C-21; Lactate; Acetylated glycoprotein; Unsaturated lipid	HDL	Increased oxidative stress; Synovial membrane degradation
Sasaki, C [31]	2019	Location: Systemic Matrix: Plasma Characteriza-tion: CE-Q-TOF-MS	Glyceric acid; Phenylalanine; Tyrosine; Pyruvic acid; Glycerol-3-phosophate; Glutamic acid; Threo-3-methyl-L-aspartic acid; Glucuronic acid; Galacturonic acid; 3-Methylhistidine; Gluconic acid; Threonic acid; Pelargonic acid; Asymmetric dimethylarginine; N,N-Dimethylglycine; Mucic acid; Glucaric acid; Lactic acid; 2-hydroxybutyric acid; 2-hydroxyisobutyric acid	Histidine; Serine; Azelaic acid; N-Acetylleucine; Cysteine-glutathione disulphide; Cysteine-glutathione disulphide (divalent); γ-butyrobetaine; 1-Methylnicotinamide	Glycolysis; TCA cycle; Amino acid metabolism; Arginine metabolism
